# Genetic effects and genotype × environment interactions govern seed oil content in *Brassica napus* L.

**DOI:** 10.1186/s12863-016-0468-0

**Published:** 2017-01-05

**Authors:** Yanli Guo, Ping Si, Nan Wang, Jing Wen, Bin Yi, Chaozhi Ma, Jinxing Tu, Jitao Zou, Tingdong Fu, Jinxiong Shen

**Affiliations:** 1 National Key Laboratory of Crop Genetic Improvement, Huazhong Agricultural University, Wuhan, 430070 China; 2 Center for Plant Genetics and Breeding, School of Plant Biology, the University of Western Australia (M080), 35 Stirling Highway, Crawley, WA 6009 Australia; 3 National Research Council Canada, Saskatoon, Saskatchewan S7N 0W9 Canada

**Keywords:** Seed oil content, Diallel, Genetic effects, *Brassica napus*

## Abstract

**Background:**

As seed oil content (OC) is a key measure of rapeseed quality, better understanding the genetic basis of OC would greatly facilitate the breeding of high-oil cultivars. Here, we investigated the components of genetic effects and genotype × environment interactions (GE) that govern OC using a full diallel set of nine parents, which represented a wide range of the Chinese rapeseed cultivars and pure lines with various OCs.

**Results:**

Our results from an embryo-cytoplasm-maternal (GoCGm) model for diploid seeds showed that OC was primarily determined by genetic effects (V_G_) and GE (V_GE_), which together accounted for 86.19% of the phenotypic variance (V_P_). GE (V_GE_) alone accounted for 51.68% of the total genetic variance, indicating the importance of GE interaction for OC. Furthermore, maternal variance explained 75.03% of the total genetic variance, embryo and cytoplasmic effects accounted for 21.02% and 3.95%, respectively. We also found that the OC of F_1_ seeds was mainly determined by maternal effect and slightly affected by xenia. Thus, the OC of rapeseed was simultaneously affected by various genetic components, including maternal, embryo, cytoplasm, xenia and GE effects. In addition, general combining ability (GCA), specific combining ability (SCA), and maternal variance had significant influence on OC. The lines H2 and H1 were good general combiners, suggesting that they would be the best parental candidates for OC improvement. Crosses H3 × M2 and H1 × M3 exhibited significant SCA, suggesting their potentials in hybrid development.

**Conclusions:**

Our study thoroughly investigated and reliably quantified various genetic factors associated with OC of rapeseed by using a full diallel and backcross and reciprocal backcross. This findings lay a foundation for future genetic studies of OC and provide guidance for breeding of high-oil rapeseed cultivars.

## Background

The seed oil content (OC) is a key measure of rapeseed quality and is also a complicated quantitative trait easily affected by the environment and difficult to investigate [1–3]. Previous studies have demonstrated that the OC of rapeseed is mainly controlled by genotype and genotype × environment interactions (GE) [4–6]; in addition, it is governed by multiple genes mainly through additive effect, and thus can be altered through breeding and selection [7–10].

A previous study on summer rapeseed has suggested that OC might be primarily controlled by maternal factors or embryo genotype or xenia [4]. The strong influence of maternal effect on the OC of F_1_ seeds is usually accompanied by weak xenia [11–13]. For maternal effect, several forms have been proposed, including the maternal inheritance of plastid, endosperm, seed coat, and maternal provision of nutrients [14, 15]. Seed lipid synthesis is independent of the leaf photosynthesis and the phloem transport of photosynthate [16], but mainly requires the supply of photosynthate from the silique wall [6, 12]. Photosynthesis of the silique wall, sugar transport in the seed coat, and the expression of fatty acid synthesis-related genes in the embryo can significantly influence the OC [17, 18]. In addition, the storage substance in seed is determined not only by the availability of assimilates (source strength), but also by the intrinsic traits of the seed (sink strength), which are controlled by the embryo genotype [19]. Therefore, variation in OC of rapeseed may be governed by multiple genetic components, including embryo, cytoplasmic, xenia, maternal, and GE effects [11–13, 20, 21].

Xenia, which represents the effect of pollen on the development and characters of seed, can be demonstrated by analyzing the differences between seeds fertilized using different pollen sources from the same plant [22, 23]. The maternal effect on OC can be investigated using reciprocal crosses [24, 25], and ancillary data from backcross progeny can be used to separate cytoplasmic effect from maternal effect [26, 27]. A significant difference between reciprocal backcrosses is strongly indicative of cytoplasmic effect. Cytoplasmic effect and maternal effect may also be distinguished by comparing reciprocal F_2_ seeds [28]. In contrast, a difference between reciprocal F_1_ hybrids that does not exist between reciprocal F_2_ hybrids would indicate that OC is determined by maternal effect without cytoplasmic effect [11].

Diallel mating designs have been frequently used to investigate the genetic effects of parents or to determine which cultivars are the best combiners for favorable alleles in hybrids. Diallel analysis provides information on the genetic behaviors of these attributes in the F_1_ generation [29]. The four methods of Griffing have usually been used to obtain genetic information on the basis of data from only one year or one location [30]; however, it has been suggested that considering multiple types of environment data could provide more reliable genetic information on the material tested [31]. The variance of general combining ability (GCA) incorporates additive epistasis, whereas that of specific combining ability (SCA) incorporates dominance epistasis [30, 32]. The observation of high additive effect for a specific trait indicates higher heritability and less environmental influence and will facilitate the selection of this trait [33]. Additive effect efficiently responds to selection, whereas non-additive effects, such as dominance and epistatic components, increase hybrid vigor in the cross combinations of cultivars. These facts suggest that the evaluation of GCA of a specific trait can guide the breeders to select the parents that can be used in breeding program for that trait, while SCA indicates the heterosis of a specific trait, and significant SCA of a cross suggests the presence of non-additive gene action [34].

In the present study, we investigated whether OC is determined by embryo, maternal, cytoplasmic, xenia or GE effects, or a combination of these factors. To this end, we estimated the components of different genetic systems and their corresponding GE. In addition, we investigated the roles of GCA, SCA, and reciprocal effect in the inheritance of OC. An improved understanding of the genetic components of OC and the combining abilities of inbred lines will help breeders to develop high-oil rapeseed cultivars and hybrids for particular geographic locations.

## Methods

### Materials and field experiments

The experiments were carried out from September of 2009 to May of 2012. Nine semi-winter rapeseed lines with differences in seed oil content (OC) were selected as parental lines from the Rapeseed Laboratory of Huazhong Agricultural University (Table 1). Three high-oil lines (HO; H1, H2, H3), three medium-oil lines (MO; M1, M2, M3), and three low-oil lines (LO; L1, L2, L3), were crossed in a 9 × 9 diallel mating scheme to produce 72 F_1_ hybrids (F_1_ seeds from maternal plants), including 36 crosses and 36 of their reciprocals in March of 2010. These 72 crosses were performed again in March of 2012, and these F_1_ seeds were only analyzed for maternal effect and xenia on the OC of rapeseed.

**Table 1:** Parents and their seed oil contents (%) in 2011 (Mean ± SE)

Lines	Parents	Source	Quality	2011
H1	8471	Pure line	High erucic acid low GSL	44.61 ± 0.55
H2	1204	Pure line	High erucic acid low GSL	47.17 ± 0.18
H3	P19	Pure line	Double Low^a^	45.46 ± 1.28
M1	Zheyou8	Registered Cultivar	High erucic acid high GSL	38.74 ± 1.36
M2	Xiangyou3	Registered Cultivar	High erucic acid high GSL	41.04 ± 0.51
M3	Huyou17	Registered Cultivar	Double Low	40.85 ± 1.13
L1	Luyou15	Registered Cultivar	Double Low	37.99 ± 0.55
L2	Luyou12	Registered Cultivar	Double Low	34.86 ± 1.20
L3	Zheng252	Registered Cultivar	Double Low	35.13 ± 0.48

*GSL* glucosinolate, *SE* standard error, *%* percentage

^a^Double low means that the content of erucic acid and total glucosinolates in seeds are below 2% and 30 mmol/g seeds, respectively

A total of 81 genetic entries (72 F_1_ hybrids from maternal plants and 9 parents) obtained from the complete diallel cross of the nine parents in May of 2010, were directly sown in the field at Huazhong Agricultural University, Wuhan, China around September 27th of both 2010 and 2011. The parental and F_1_ seeds were planted in a randomized block design with three replications over two consecutive growing seasons, and each block contained two rows with 10 plants in each row at a space of 30 cm × 15 cm. The OC of self-pollinated parents and F_2_ generation (F_2_ seeds from F_1_ plants) plants was calculated as the mean value of 20–56 individually harvested plants in both 2011and 2012, and that of the open-pollinated F_2_ generation was calculated as the mean value of 13–50 individually harvested plants in 2011.

Environmental factors, such as temperature, light, and weather during seed development and difference in flowering time, could influence OC and the results of the study. Therefore, since F_1_ plants have the same developmental timing, backcross using an F_1_ plant as the maternal parent could be used to solve these problems [27]. HO (H2, H3) and LO (L1, L2) were selected for producing backcross (BC) and reciprocal backcross (RBC) generations. The four parents and their eight F_1_ hybrids produced in March of 2010 were directly sown in the field at Huazhong Agricultural University, Wuhan, China around September 27th of both 2010 and 2011. In the spring of both 2011 and 2012, a total of 32 BC and RBC combinations were produced, including eight BC_1_s, eight BC_2_s (with F_1_ plants as female parents), eight RBC_1_s, and eight RBC_2_s (with F_1_ plants as male parents), and each of the combinations was repeated three times and used to study the maternal, xenia and cytoplasmic effects on OC.

All plants were isolated by paper bags at the beginning of flowering to ensure self-pollination of parents and F_2_ seeds, and the bags were removed at the end of full flowering. All plants were harvested and threshed at early May in 2010, 2011 and 2012. The total OC was measured using the Foss NIR-Systems 5000 near-infrared reflectance spectroscope (NIR-Systems, Inc., Silver Spring, MD, USA) [35], with the parameters described by Gan et al. [36].

### Statistical analysis

Data were analyzed using a general linear model (GLM) [37]. Based on the GLM, least-square means were used to compute the combining ability based on Griffing’s diallel analysis with Method 1 (full diallel set), Model 1 [30]. All variance analyses and combining ability (GCA, SCA, reciprocal) estimation were performed using the SAS codes published by Zhang and Kang [31]. On the basis of Cong [38] and Duan et al. [39], the method proposed by Wang et al. [11] was used to estimate the genetic components of maternal effect and xenia in rapeseed. The OC of F_1_ seeds was calculated as F_1_ = MP_1_ + XP_2_, where M was the value of maternal effect and X was the value of xenia (X = 1–M).

The partitions of embryo, maternal, cytoplasmic effects as well as the corresponding GE were estimated using the embryo-cytoplasm-maternal (GoCGm) model [40, 41] for diploid seeds in QGAStation1.0 (http://ibi.zju.edu.cn/software/qga/index.htm). The genetic variance components were estimated using the minimum norm quadratic unbiased estimation (MINQUE) (0/1) method [42], and the genetic effects of each parent were investigated using the adjusted unbiased prediction (AUP) method [43]. Standard errors of the estimated variance and predicted genetic effects were then analyzed using a Jackknife procedure [44], and t-tests were used to test for significant differences in the traits examined.

## Results

### Phenotypic variation

We found that the self-pollinated seeds from nine parents, including HO (H1, H2, and H3), MO (M1, M2, and M3), and LO (L1, L2, and L3), exhibited significant differences in OC, and that the differences between the parents with the highest and lowest OC in each year was about 12.00% (Tables 1, 2). The OC of the 72 F_1_ hybrids was strongly influenced by the maternal parent. In 2012, the mean OC of the eight F_1_ lines with H1 as the female parent was 42.01%, which was comparable to that of H1 (42.79%), whereas that of the eight F_1_ lines with H1 as the male parent was 38.04% (Table 2). Similarly, the mean OC of the eight F_1_ lines with L3 as the female parent was 32.44%, which was comparable to that of L3 (29.63%), and that of the eight F_1_ lines with L3 as the male parent was 37.10%, which was much higher than that of L3. Similar trends were observed in the other sets of crosses in both 2010 and 2012. Thus, the OC of F_1_ hybrids was similar to that of maternal parent, even with different male parents.

**Table 2:** Oil contents (%) of the complete diallel crosses F_1_ seeds harvested in 2010 and 2012 (Mean ± SE)

Lines	2010			2012		
	Self-pollination	F_1(F)_^ a^	F_1(M)_^ b^	Self-pollination	F_1(F)_^ a^	F_1(M)_^ b^
H1	49.21 ± 1.42	48.35 ± 1.54	40.00 ± 4.35	42.79 ± 0.40	42.01 ± 2.16	38.04 ± 6.09
H2	49.26 ± 1.62	49.43 ± 1.24	42.08 ± 3.63	45.80 ± 1.83	44.65 ± 2.06	40.19 ± 3.50
H3	42.20 ± 2.20	39.42 ± 3.69	40.74 ± 5.64	40.99 ± 1.36	42.05 ± 1.65	37.08 ± 5.12
M1	40.66 ± 0.45	39.87 ± 2.03	41.50 ± 5.78	37.37 ± 1.93	38.17 ± 2.19	39.11 ± 5.22
M2	40.03 ± 0.45	38.50 ± 1.92	42.11 ± 5.69	39.43 ± 1.60	39.82 ± 1.76	39.49 ± 4.89
M3	36.81 ± 0.43	38.70 ± 1.95	39.76 ± 4.41	37.00 ± 2.50	38.40 ± 1.66	38.14 ± 5.32
L1	37.84 ± 2.65	39.00 ± 2.63	40.26 ± 4.80	35.27 ± 3.53	36.16 ± 3.50	37.27 ± 6.15
L2	35.73 ± 1.67	35.75 ± 2.48	40.66 ± 5.39	27.26 ± 3.70	30.55 ± 4.18	39.48 ± 4.44
L3	36.50 ± 0.67	38.85 ± 1.28	39.54 ± 5.27	29.63 ± 0.84	32.44 ± 3.70	37.10 ± 4.91

*%* percentage, *SE* standard error

^a^Mean value of the F_1_ derived from the line as female crossed with other 8 lines

^b^Mean value of the F_1_ derived from the line as male crossed with other 8 lines

**Fig. 1:**
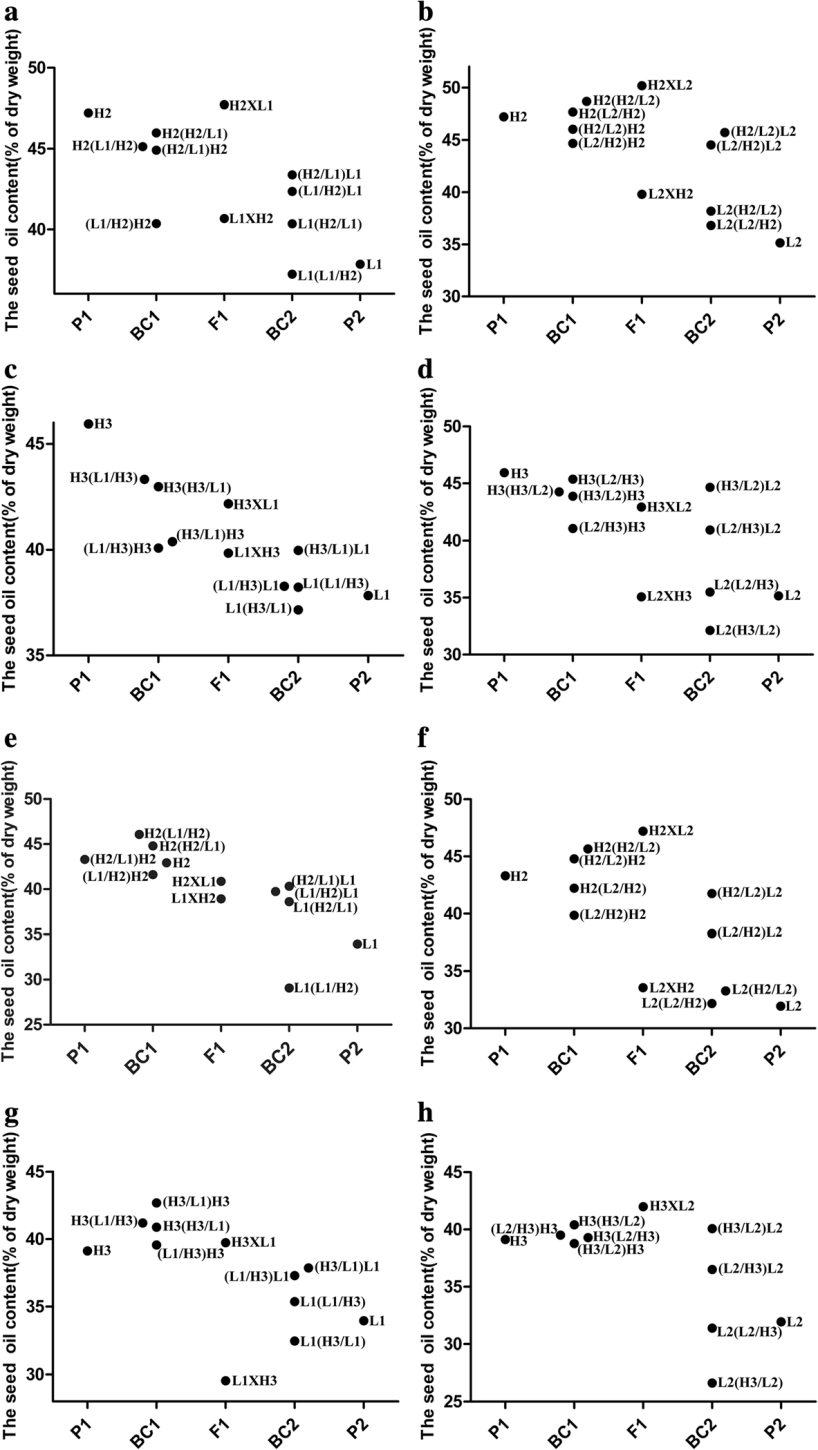
Average values of seed oil content (%) in 4 parents and 10 reciprocal generations within 6 cross-combinations. **a**, **e** The mean seed oil contents of H2, L1 and reciprocal F_1_, BC_1_ and BC_2_ combinations were tested in 2011 and 2012, respectively. **b**, **f** The mean seed oil contents of H2, L2 and reciprocal F_1_, BC_1_ and BC_2_ combinations were tested in 2011 and 2012, respectively. **c**, **g** The mean seed oil contents of H3, L1 and reciprocal F_1_, BC_1_ and BC_2_ combinations were tested in 2011 and 2012, respectively. **d**, **h** The mean seed oil contents of H3, L2 and reciprocal F_1_, BC_1_ and BC_2_ combinations were tested in 2011 and 2012, respectively

The results in Table 3 showed that the mean OC of open-pollinated F_2_ seeds was about 2% higher than that of self-pollinated F_2_ seeds, and the mean values for most of the F_2_ seeds from reciprocal crosses were more or less similar, despite a small amount of inbreeding depression. In addition, differences between the F_1_ reciprocal crosses disappeared in the F_2_ generation for most combinations, thus demonstrating a maternal effect and little or no cytoplasmic effect (Tables 2, 3). The analysis of backcrosses confirmed the predominant influence of maternal parent on OC, even with the elimination of differences in flowering time (Fig. 1). Furthermore, the mean OCs of (H2/L1)H2 and (H2/L1)L1 were similar (Fig. 1a, e), and it was the same case for (L1/H2)H2 and (L1/H2)L1 (Fig. 1a, e). However, when H2 was used as the maternal parent to cross with four F_1_ hybrid lines (L1/H2, H2/L1, H2/L2, L2/H2), the various backcrosses exhibited different OCs, indicating the presence of a slight xenia (Fig. 1a, b, e, f). Similar trends were observed in other three sets of backcrosses, confirming that maternal effect was the main determinant of OC and that the influence of pollen source was minor (Fig. 1).

**Table 3:** Oil contents (%) of F_2_ seeds harvested from F_1_ plants grown in the field in 2011 (Mean ± SE)

Crosses	Self-pollination		Open-pollination	
	Crosses	Reciprocal	Crosses	Reciprocal
H1 × H2	44.24 ± 1.69(32)^a^	45.71 ± 2.17(40)	46.31 ± 1.61(24)	47.81 ± 2.00(34)
H1 × H3	44.68 ± 2.36(37)	43.98 ± 2.13(47)	46.76 ± 1.66(28)	45.06 ± 2.36(33)
H1 × M1	44.12 ± 2.48(45)	42.61 ± 2.18(50)	45.35 ± 2.34(32)	44.58 ± 2.07(45)
H1 × M2	44.36 ± 1.73(48)	44.08 ± 1.85(48)	45.99 ± 1.29(35)	45.73 ± 1.44(41)
H1 × M3	45.41 ± 2.69(44)	43.57 ± 3.44(32)	46.84 ± 2.46(30)	45.63 ± 1.93(31)
H1 × L1	43.74 ± 2.33(41)	44.34 ± 2.81(54)	45.22 ± 1.88(25)	45.79 ± 2.02(35)
H1 × L2	43.92 ± 2.99(48)	43.15 ± 2.10(43)	45.35 ± 2.65(24)	44.69 ± 2.05(23)
H1 × L3	42.23 ± 2.12(38)	42.44 ± 2.16(51)	45.53 ± 1.71(25)	45.01 ± 1.93(29)
H2 × H3	46.89 ± 1.53(48)	46.61 ± 1.66(54)	49.25 ± 1.47(41)	48.23 ± 1.80(49)
H2 × M1	43.58 ± 1.71(51)	43.29 ± 1.93(48)	45.94 ± 1.39(41)	45.55 ± 1.50(44)
H2 × M2	46.92 ± 1.45(50)	46.82 ± 1.89(46)	48.84 ± 1.27(40)	48.30 ± 2.02(36)
H2 × M3	42.08 ± 2.36(47)	41.96 ± 2.29(42)	44.48 ± 1.56(31)	44.57 ± 1.37(32)
H2 × L1	42.73 ± 2.21(47)	43.09 ± 2.68(52)	45.37 ± 1.15(36)	45.03 ± 2.01(36)
H2 × L2	44.26 ± 1.82(49)	44.52 ± 2.20(56)	46.35 ± 1.57(28)	46.31 ± 1.99(33)
H2 × L3	40.76 ± 2.64(42)	42.53 ± 2.09(49)	42.83 ± 2.17(27)	44.18 ± 1.97(35)
H3 × M1	40.27 ± 1.70(52)	41.14 ± 2.30(38)	42.59 ± 1.53(43)	43.16 ± 1.61(31)
H3 × M2	45.97 ± 1.81(51)	46.24 ± 1.76(45)	47.67 ± 1.63(46)	48.50 ± 1.33(36)
H3 × M3	39.98 ± 2.32(49)	42.17 ± 1.77(31)	42.52 ± 2.54(33)	44.54 ± 0.98(21)
H3 × L1	40.47 ± 1.63(55)	41.22 ± 2.01(48)	42.00 ± 1.60(43)	43.26 ± 1.78(34)
H3 × L2	41.58 ± 1.57(42)	41.48 ± 1.83(29)	43.08 ± 1.56(35)	44.22 ± 2.01(18)
H3 × L3	39.12 ± 1.93(48)	39.38 ± 2.65(49)	41.69 ± 1.87(38)	41.68 ± 2.42(36)
M1 × M2	40.99 ± 2.27(41)	41.48 ± 1.46(54)	42.41 ± 2.14(34)	43.47 ± 1.10(48)
M1 × M3	40.62 ± 2.17(55)	40.90 ± 2.00(47)	42.66 ± 1.74(50)	43.01 ± 1.44(38)
M1 × L1	38.75 ± 2.83(23)	41.74 ± 1.59(36)	39.81 ± 1.44(13)	43.35 ± 1.67(30)
M1 × L2	40.48 ± 1.96(37)	39.19 ± 2.60(51)	42.85 ± 1.32(28)	41.40 ± 2.60(36)
M1 × L3	37.86 ± 1.99(51)	38.16 ± 2.36(48)	40.15 ± 1.60(40)	41.36 ± 1.64(42)
M2 × M3	41.24 ± 1.49(50)	41.66 ± 1.25(46)	43.02 ± 1.37(41)	43.29 ± 1.26(47)
M2 × L1	42.02 ± 1.37(53)	41.47 ± 1.65(34)	43.23 ± 1.69(43)	43.18 ± 1.19(30)
M2 × L2	40.03 ± 1.73(53)	39.58 ± 3.19(41)	41.59 ± 1.47(30)	42.06 ± 2.79(34)
M2 × L3	38.71 ± 2.04(44)	39.33 ± 1.64(53)	40.52 ± 1.74(33)	41.54 ± 1.20(36)
M3 × L1	39.59 ± 2.72(46)	39.76 ± 3.08(48)	41.03 ± 2.21(36)	41.67 ± 1.85(38)
M3 × L2	39.09 ± 2.34(44)	39.54 ± 2.06(33)	41.18 ± 2.12(26)	41.88 ± 1.08(14)
M3 × L3	37.61 ± 2.44(46)	37.11 ± 2.51(47)	40.96 ± 1.54(39)	40.36 ± 2.15(39)
L1 × L2	38.90 ± 2.15(50)	37.88 ± 2.43(48)	39.45 ± 2.25(33)	40.08 ± 1.75(32)
L1 × L3	37.85 ± 1.81(47)	37.58 ± 2.28(55)	40.52 ± 1.36(29)	40.31 ± 1.58(34)
L2 × L3	37.58 ± 2.11(45)	36.72 ± 2.02(47)	40.05 ± 2.62(25)	39.74 ± 1.77(30)

*%* percentage*, SE* standard error

^a^plant number of F_1_ plants

### Variance analysis of means and gene action

A combined analysis of variance also indicated significant differences in OC among genotypes (Table 4). The significant mean squares of GE for OC indicated that the magnitude of the trait in different genotypes varied over the two years, and the differences between plants in different blocks were also significant, indicating that OC was easily affected by the environment. GCA and SCA also significantly contributed to the OC variation over the years, indicating the importance of both additive and non-additive effects on OC (Table 4). Further partitioning of reciprocal sum squares indicated that maternal effect was significant, whereas non-maternal effect was not, suggesting that OC was not under strict nuclear control and could also be influenced by cytoplasm. The GCA × Environment showed significant effect on OC, suggesting that the additive variances were influenced by the environment. However, the SCA × Environment did not show any effect. Therefore, the effect of environment on non-additive components of genetic variance was not significant.

**Table 4:** 9 × 9 diallel analysis of variance for the oil content of rapeseed (Griffing’s Method 1)

Sources	DF	Sum of squares	Mean squares	F-value	P > F
Environment (E)	1	1447.84	1447.84^**^	118.68	0.000
Block	4	48.80	12.20^**^	13.87	0.000
Genotype (G)	80	4522.28	56.53^**^	19.84	0.000
GCA	8	3931.52	491.44^**^	40.41	0.000
SCA	27	304.96	11.29^**^	8.55	0.000
REC	36	56.01	1.56	0.94	0.540
M	8	26.58	3.32^**^	3.49	0.040
NM	28	29.42	1.05	0.56	0.920
G × E	80	228.00	2.85^**^	3.24	0.000
GCA × E	8	97.29	12.16^**^	13.82	0.000
SCA × E	27	35.55	1.32	1.50	0.730
REC × E	36	59.77	1.66	1.89	0.430
M × E	8	7.56	0.95	1.08	0.790
NM × E	28	52.20	1.86	2.11	0.270
Error	320	281.00	0.88		

*DF* degree of freedom, *GCA* general combining ability, *M* maternal, *NM* non-maternal component, *REC* reciprocal

*SCA* specific combining ability

**indicate significance at the 0.01 level

### Combining ability

The GCA was significant for all parents even though some parents had positive values and other had negative values, whereas no parental line exhibited significant maternal effect (Table 5). H2 (3.21^**^) and H1 (2.87^**^) had the highest GCA for OC, whereas L3 (−3.06^**^) and L2 (−2.06^**^) were negative combiners with reduced OC in F_1_. The SCA in several crosses was significant, and reciprocal effect was only significant in two crosses. Lines H3 and M2 showed significantly positive GCA, whereas M1, M3 and L1 exhibited significantly negative GCA. Crosses H1 × M3, H1 × L1, H1 × L3, H2 × H3, H2 × M2 and H3 × M2 exhibited significantly positive SCA and were the best specific combiners for improvement of OC (Table 5), The negative SCA of H1 × H2, H1 × H3, H1 × M2, H3 × M1 and L2 × L3 observed with lower OC suggested that they were poor parental combinations for breeding. The reciprocal effect was estimated to be not significant in most reciprocal crosses, except for the reciprocals of L3 × M3 and L1 × H3. The cross L3 × M3 showed significant positive reciprocal effect indicating a negative cytoplasmic effect from the maternal parent L3. The negative reciprocal effect of L1 × H3 indicated a positive cytoplasmic effect from maternal parent L1. Therefore, L3 and L1 should be respectively exploited as male and maternal parent in crosses for OC improvement.

**Table 5:** Estimates of GCA, SCA, reciprocal effect and maternal effect (Griffing’s Method 1)

	H1	H2	H3	M1	M2	M3	L1	L2	L3	GCA	Maternal
H1		−2.30^b^	−0.92^b^	0.32	−1.13^b^	1.26^b^	0.99^b^	0.45	1.32^b^	2.87^b^	0.11
H2	−0.24		0.84^b^	−0.22	0.87^b^	−0.11	−0.41	0.68^a^	0.64^a^	3.21^b^	0.03
H3	0.18	0.41		−1.18^b^	2.30^b^	−0.65^a^	−0.53	−0.02	0.16	0.73^b^	−0.21
M1	0.41	−0.11	0.47		−0.60	0.66^a^	0.33	0.46	0.22	−1.00^b^	−0.14
M2	0.19	0.02	−0.24	−0.20		−0.53	0.18	−0.78^a^	−0.31	1.05^b^	0.03
M3	0.36	0.15	−0.29	−0.14	−0.37		−0.38	0.04	−0.30	−0.65^b^	0.20
L1	−0.33	−0.25	−0.79^a^	−0.43	−0.02	0.18		0.36	−0.55	−1.08^b^	0.27
L2	0.23	−0.03	−0.11	0.16	0.05	0.13	0.58		−1.19^b^	−2.06^b^	−0.05
L3	0.20	−0.16	−0.39	0.05	0.33	1.16^b^	0.24	0.53		−3.06^b^	−0.22

*GCA* general combining ability, *SCA* specific combining ability

^a^,^b^Significantly different from zero at the 0.05 and 0.01 probability levels, respectively

### Maternal effect and xenia

Based on the 54 F_1_ hybrids (HO × MO, HO × LO, MO × HO, MO × LO, LO × HO, LO × MO) harvested in 2010 and 2012, the mean estimated value of maternal effect was 0.84 in 2010 and 0.89 in 2012, and that of xenia was 0.16 in 2010 and 0.11 in 2012 (Table 6). On average across the two years, maternal effect accounted for 0.87 of the observed variation, whereas xenia accounted for only 0.13 of it, confirming a very strong maternal effect on the OC of F_1_ seeds and a weak effect of xenia. Some crosses (H1 × MO and M3 × LO) exhibited values of maternal effect around 1 consistently across the two years, suggesting 100% maternal influence. Some crosses (H3 × LO and M1 × LO) showed maternal effect and xenia varying markedly between two years and also had large standard errors (SE), suggesting that these crosses had relatively weak maternal effects.

**Table 6:** Estimated values of maternal effect and xenia on F_1_ hybrid oil contents in 2010 and 2012

Female	Crosses	2010		2012	
		Maternal (M)	Xenia (X)	Maternal (M)	Xenia (X)
H1	H1 × LO	0.81 ± 0.11	0.09 ± 0.11	0.86 ± 0.18	0.14 ± 0.18
	H1 × MO	1.03 ± 0.01	−0.03 ± 0.01	1.12 ± 0.19	−0.12 ± 0.19
H2	H2 × LO	0.96 ± 0.10	0.04 ± 0.10	1.04 ± 0.08	0.09 ± 0.14
	H2 × MO	1.17 ± 0.02^a^	−0.17 ± 0.02	0.95 ± 0.27	0.05 ± 0.27
H3	H3 × LO	0.42 ± 0.16	0.58 ± 0.16	0.82 ± 0.17	0.18 ± 0.17
	H3 × MO	0.84 ± 0.93	0.16 ± 0.93	1.06 ± 0.27	−0.06 ± 0.27
M1	M1 × HO	0.81 ± 0.20	0.19 ± 0.20	0.82 ± 0.22	0.18 ± 0.22
	M1 × LO	0.59 ± 0.69	0.41 ± 0.69	1.09 ± 0.54	−0.09 ± 0.54
M2	M2 × HO	1.58 ± 0.86	−0.58 ± 0.86	0.89 ± 0.19	0.11 ± 0.19
	M2 × LO	0.70 ± 0.64	0.30 ± 0.64	1.02 ± 0.10	−0.02 ± 0.10
M3	M3 × HO	0.59 ± 0.39	0.41 ± 0.39	0.74 ± 0.26	0.26 ± 0.26
	M3 × LO	1.16 ± 3.12	−0.16 ± 3.12	1.17 ± 0.27	−0.17 ± 0.27
L1	L1 × HO	0.80 ± 0.37	0.20 ± 0.37	0.89 ± 0.22	0.11 ± 0.22
	L1 × MO	0.66 ± 1.02	0.34 ± 1.02	0.76 ± 0.75	0.24 ± 0.75
L2	L2 × HO	0.74 ± 0.06	0.26 ± 0.06	0.75 ± 0.12	0.25 ± 0.12
	L2 × MO	1.24 ± 0.33	−0.24 ± 0.33	0.74 ± 0.14	0.26 ± 0.14
L3	L3 × HO	0.70 ± 0.04	0.30 ± 0.04	0.89 ± 0.39	0.11 ± 0.39
	L3 × MO	0.35 ± 0.10	0.65 ± 0.10	0.49 ± 0.42	0.51 ± 0.42
Mean		0.84	0.16	0.89	0.11

^a^The maternal effect >1 was primarily due to the existence of ultra-high or ultra-low oil content parental individuals

### Cytoplasmic effect

Analyses of backcrosses revealed the minor or negligible influence of cytoplasmic effect, although the backcrosses also displayed some variations between 2011 and 2012 (Fig. 1), which demonstrated that environment might have some influence on cytoplasmic effect. In 2011, for example, the average OC of (H3/L2)H3 was higher than that of (L2/H3)H3, and that of (H3/L2)L2 was higher than that of (L2/H3)L2 as well. However, in 2012, the mean OC of (H3/L2)L2 was higher than that of (L2/H3)L2, which might be owing to positive cytoplasmic effect, whereas that of (H3/L2)H3 was lower than that of (L2/H3)H3, which might be due to negative cytoplasmic effect. The mean OC of (H2/L2)L2 was higher than that of (L2/H2)L2, and that of (H2/L2)H2 was higher than that of (L2/H2)H2, which could be attributed to the positive cytoplasmic effect (Fig. 1b, f). The average OC of (H2/L1)H2 was higher than that of (L1/H2)H2, whereas that of (H2/L1)L1 was similar to that of (L1/H2)L1 in both 2011 and 2012. In contrast, the mean OCs of (H3/L1)H3 and (L1/H3)H3 were comparable, and it was the same case for (H3/L1)L1 and (L1/H3)L1, which again suggested the lack of cytoplasmic effect.

### Components of total genetic variance

The results from GoCGm model showed that 13.81% of the phenotypic variance (V_P_) in OC could be attributed to environmental variations and experimental errors, whereas the rest (86.19%) was attributable to genetic (V_G_) and GE (V_GE_) components (Table 7). Genetic and GE variance components respectively accounted for 48.32% and 51.68% of the total genetic variance (V_G_ + V_GE_). Embryo additive (V_A_), embryo additive interaction (V_AE_), maternal additive (V_Am_) and maternal dominant interaction (V_DmE_) variance components were significant, whereas embryo dominant interaction (V_DE_), cytoplasm interaction (V_CE_), maternal additive (V_AmE_), and maternal dominant (V_Dm_) variance components were not. Maternal variances (V_Am_ + V_Dm_ + V_AmE_ + V_DmE_) explained 75.03% of the total genetic variance, whereas embryo and cytoplasmic effects accounted for only 21.02% and 3.95% of it, respectively. Overall, these results indicated that OC was predominantly influenced by maternal effect, followed by embryo and cytoplasmic effects.

**Table 7:** Estimation of genetic variance components of the seed oil content in rapeseed

Parameter	Variance	Parameters	Variance	Parameter	Variance (%)
*V*_*A*_	0.048^a^	*V*_*AE*_	0.047^a^	*V*_*G*_ *+ V*_*GE*_ */V*_*P*_	86.19
*V*_*D*_	0.086	*V*_*DE*_	0.000	*V*_*e*_ */V*_*P*_	13.81
*V*_*C*_	0.034	*V*_*CE*_	0.000	*V*_*GE*_ */V*_*G*_ *+ V*_*GE*_	51.68
*V*_*Am*_	0.248^a^	*V*_*AmE*_	0.000	*V*_*G*_ */V*_*G*_ *+ V*_*GE*_	48.32
*V*_*Dm*_	0.000	*V*_*DmE*_	0.398^a^	*V*_*A*_ *+ V*_*D*_ *+ V*_*AE*_ *+ V*_*DE*_ */V*_*G*_ *+ V*_*GE*_	21.02
		*V*_*e*_	0.138^a^	*V*_*C*_ *+ V*_*CE*_ */V*_*G*_ *+ V*_*GE*_	3.95
				*V*_*Am*_ *+ V*_*Dm*_ *+ V*_*AmE*_ *+ V*_*DmE*_ */V*_*G*_ *+ V*_*GE*_	75.03

^a^indicates significance at the 0.01 level, *%* percentage

### Estimation of genetic components of parents

H2 showed the highest additive effect, whereas L3 displayed the lowest additive effect, and we also found that H1, H2, H3, M1 and L1 exhibited significant positive maternal additive effect, whereas other parents showed significant negative maternal additive effect (Table 8). L2 exhibited the lowest maternal additive effect, whereas H1 showed the highest maternal additive effect. Cytoplasmic effects were positive in H1, H2, M1, M3 and L1, whereas the cytoplasmic effects of the remaining four lines were insignificantly or significantly negative. According to our estimated values, H1 exhibited the highest cytoplasmic effect (1.79 ± 0.86) among the nine parents, indicating the high probability of generating high-oil rapeseed with this line being used as the maternal parent. In addition, H2, M1, M2, and L2 displayed negative homozygous dominance, whereas the remaining five lines exhibited positive homozygous dominance (Table 8). L3 showed the highest homozygous dominance effect, indicating that it is more likely produce high heterosis. Moreover, all of the genetic effects of H1 were positive, suggesting that it might be an ideal maternal parent for breeding high-oil rapeseed.

**Table 8:** Estimation of genetic effects for seed oil content in rapeseed

Lines	Cytoplasm effect	Additive effect	Maternal Additive	Dominance
H1	1.79 ± 0.86^a^	0.80 ± 0.06^b^	2.88 ± 0.13^b^	3.50 ± 1.00^b^
H2	1.08 ± 0.24^b^	1.68 ± 0.09^b^	1.32 ± 0.05^b^	−2.18 ± 0.62^b^
H3	−0.09 ± 0.02^b^	−0.37 ± 0.02^b^	2.25 ± 0.09^b^	0.45 ± 0.04^b^
M1	0.16 ± 0.03^b^	0.75 ± 0.03^b^	0.94 ± 0.05^b^	−4.94 ± 2.24^a^
M2	−0.39 ± 0.03^b^	0.68 ± 0.05^b^	−0.09 ± 0.00^b^	−0.69 ± 0.45
M3	0.10 ± 0.03^b^	0.02 ± 0.01^b^	−1.52 ± 0.07^b^	2.39 ± 0.28^b^
L1	0.49 ± 0.24^a^	−1.09 ± 0.03^b^	0.16 ± 0.01^b^	0.75 ± 0.04^b^
L2	−1.19 ± 0.42^b^	0.17 ± 0.06^b^	−3.48 ± 0.17^b^	−0.50 ± 0.07^b^
L3	−1.96 ± 1.88	−1.14 ± 0.03^b^	−2.46 ± 0.14^b^	4.33 ± 0.28^b^

^a^significant at 0.05, ^b^significant at 0.01

## Discussion

Investigation of the influence of different genetic systems can facilitate a better understanding of the nature of gene interactions that could influence OC. Our study demonstrates that the variation of OC is mainly determined by genetic and GE components (Table 7), though the environment also has a significant influence [7, 8]. However, the influence of GE should not be neglected, since GE accounted for 51.68% of the total genetic variance. Significant interactions have been observed between male or female and the environment [13]. GE is a critical factor for developing varieties with wide geographical adaptability and should be taken into account in genetic model and breeding process. Data from the genetic model clearly demonstrated that OC was mainly controlled by the maternal parent, with the maternal effect accounting for 75% of the genetic variance. Embryo effect accounted for 21% of the genetic variance and cytoplasmic effect was detectable only at a very low level (Table 7). The present study confirms the earlier findings that the OC of F_1_ seeds is mainly controlled by maternal effect, and xenia is weak [11, 13, 45]. Analysis of reciprocal backcrosses demonstrates that maternal, xenia, and cytoplasmic effect can all influence OC (Fig. 1). Therefore, our genetic analysis confirms that OC is determined by maternal, embryo, cytoplasm, xenia and GE effects.

Previous studies have shown that the photosynthesis in silique wall makes a crucial contribution to OC [6, 12, 16, 17], providing an explanation for the influence of maternal parent on OC. Therefore, the selection for high-oil rapeseed would be more effective based on the photosynthetic activity of silique wall than based on the performance of maternal parent. It should be noted that xenia has a direct genetic effect on OC in many crops [11, 12, 46, 47], and is applied not only in genetic and physiological research but also in crop breeding and production. Similar to the case of corn and soybean, controlling the pollen source and considering xenia are required in both breeding programs and investigation of OC in rapeseed. Therefore, decision on parents and cross direction are very important for hybrid rapeseed breeding and production.

The OC of self-pollinated seeds was lower than that of open-pollinated seeds harvested from the same plant in the F_2_ generation (Table 3). This result is similar to the finding of Hom et al. [48]. Earlier studies reported that the OC of F_1_ seeds was significantly lower than that of self-pollinated seeds when a HO female was crossed with a LO male, but it was significantly higher than that of self-pollinated seeds when a LO female was crossed with a HO male [11, 12]. Similar results were obtained for soybean [49]. Although xenia has a direct genetic effect on OC of rapeseed [11, 12, 45], different pollens may not actually be responsible for the difference in OC between self-pollinated and open-pollinated seeds. During seed development, self-pollinated seeds were isolated using paper bags, whereas open-pollinated seeds were not. Thus, the difference in microclimate may have influenced the OC. Concurrently elevated CO_2_ and temperature could reduce seed biomass by half and further reduce losses in fatty acids and OC [50]. Seeds grown in high-light environments tend to have higher OC [51]. Hua et al. [12] suggested that local and tissue-specific photosynthetic activity in the silique wall were the main determinant for OC, and factors of weather and temperature could also influence photosynthesis of the silique wall, thereby affecting OC. Thus, the influence of microclimate on the photosynthesis of silique wall might explain the lower OC of self-pollinated seeds.

Our analysis of combining ability indicated the presence of both additive and non-additive gene actions in the parental lines (Table 4). Additive effect is equivalent to GCA, parental lines with high GCA for a specific trait may be better candidates as parental lines [30, 32, 34, 52]. Lines that had positive additive and maternal additive effects, such as H1, H2 and M1, are desirable general combiners that can be used for OC improvement. Cytoplasmic genes can persist through generations and are also expressed as additive effect [52]. Therefore, lines with positive cytoplasmic effect, such as H1, H2 and L1, could be used as maternal parents. It is evident that H1 and H2 are good combiners and that the genotypes might be the best candidates as maternal parents for improvement of OC. Hybrids in both *pol* and *mur* cytoplasmic male sterility systems exhibited significantly lower OC (by 1.3% and 1.4%, respectively) than identical hybrids constructed in the cytoplasm (*nap*) common in rapeseed [53, 54]. Line H1, which had positive additive, maternal additive, homozygous maternal dominance, and cytoplasmic effects, would be a more effective parent for high-oil rapeseed breeding. Crosses such as H3 × M2 and H1 × M3, which exhibited significant SCA, could be used in the development of hybrid varieties.

## Conclusion

Based on analyses of a 9 × 9 full diallel scheme and selected backcross and reciprocal backcross, we concluded that the OC is simultaneously controlled by genetic components of maternal, embryo, xenia, cytoplasmic, and GE effects in rapeseed. Maternal effect is the most important factor, accounting for 75% of the genetic variance, followed by embryo effect, which accounts for 21% of the genetic variance. Therefore, selection of maternal parents is paramount in the genetic improvement of OC in rapeseed. Together with previous studies, additional information regarding the role of genetic components in determining OC could help breeder to better manipulate the OC of rapeseed.

## Abbreviations

AUP: Adjusted unbiased prediction
BC: Backcross
GCA: General combining ability
GE: Genotype × environment interactions
HO: High-oil lines
LO: Low-oil lines
MINQUE: Minimum norm quadratic unbiased estimation
MO: Medium-oil lines
NIRS: Near infrared reflectance spectroscopy
OC: Seed oil content
RBC: Reciprocal backcross
REC: Reciprocal effect
SCA: Specific combining ability
SE: Standard error
